# Emmetropic eye growth in East Asians and non-East Asians

**DOI:** 10.1111/opo.13195

**Published:** 2023-06-27

**Authors:** Fabian SL Yii

**Affiliations:** 1 Centre for Clinical Brain Sciences, University of Edinburgh, Chancellor's Building, 49 Little France Crescent, Edinburgh, EH16 4SB UK; 2 Curle Ophthalmology Laboratory, Institute for Regeneration and Repair, University of Edinburgh, Edinburgh, UK

**Keywords:** axial elongation rate, axial length, emmetropia, ethnicity, eye growth, myopia

## Abstract

**Purpose:**

To compare axial length (AL) growth curves in East Asian (EA) and non-EA emmetropes.

**Methods:**

A meta-regression of 28 studies with emmetrope-specific AL data (measured with optical biometry) was performed. Emmetropia was defined as spherical equivalent refraction (SER) between −0.50 and +1.25 D, determined under cycloplegia if the mean age was ≤20 years. The AL growth curve (mean AL vs. mean age) was first fitted to the full dataset using a weighted nonlinear mixed-effects model, before refitting the model with ethnicity as a two-level grouping variable (EA vs. non-EA). Ethnic differences in growth curve parameters were tested using the Wald test.

**Results:**

A total of 3331 EA and 1071 non-EA emmetropes (mean age: 6.5–23.1 years) were included. There was no evidence of an ethnic difference in either final AL (difference: 0.15 mm, 95% CI: −0.04 to 0.35 mm, *p* = 0.15) or initial AL, as represented by the amount that the final AL needed to be offset to obtain the *y*-intercept (difference: −2.77 mm, 95% CI: −10.97 to 5.44, *p* = 0.51). Likewise, AL growth rate (curve steepness) did not differ between ethnic groups (difference: 0.09, 95% CI: −0.13 to 0.31, *p* = 0.43). Collectively, AL growth rate decreased from 0.24 mm/year at 6 years of age to around 0.05 mm/year at 11 years of age, after which it dipped below the repeatability of optical biometry (±0.04 mm) and practically plateaued around 16 years of age (final AL: 23.60 mm).

**Conclusions:**

EA and non-EA emmetropes have comparable AL growth curves.

**Supplementary Information:**

The online version of this article (doi:10.1111/opo.13195) contains supplementary material, which is available to authorized users.

## Key points

- East Asian emmetropes (mostly ethnic Chinese) do not undergo faster axial growth than their counterparts from outside East Asia (mostly Caucasians).
- There is no evidence that axial length differs between East Asian and non-East Asian emmetropes aged 6 years and above.
- Across ethnic groups, normal emmetropes undergo decelerated axial growth from 6 to 11 years of age (from 0.24 to 0.05 mm/year), after which the growth rate is of little practical importance.

## BACKGROUND

Sorsby^1^ recognised as far back as 1933 that, with improved mechanistic understanding of normal eye growth, one might be able to ‘look into the seeds of time’ and predict myopia development. Earlier longitudinal studies that shed light on how AL changes with age in emmetropic children, albeit scarce, are valuable on that score.^2,3^ More recently, Chen et al.^4^ and Rozema^5^ employed meta-regression to pool normative ocular biometric data from predominantly cross-sectional studies, further adding to the growing body of knowledge on normal eye growth.

However, some gaps remain to be addressed. First, the employment of ocular ultrasonography by previous longitudinal studies^2,3^ and several primary studies included in the meta-regression^4,5^ described above limited the precision of AL estimates in these works. Compared with optical biometry, ocular ultrasonography has significantly lower repeatability in normal children (−0.85 to +0.67 mm vs. −0.05 to +0.04 mm).^6^ Moreover, no work has directly investigated whether AL growth curves differ between East Asian (EA) and non-EA emmetropes above 6 years of age, a period characterised by increased susceptibility to myopia development.^7^ In light of this, the present work aimed to derive and compare ethnic-specific AL growth curves in emmetropes by synthesising data from primary studies that employed optical biometry.

## METHODS

For inclusion, studies must have used non-contact optical biometry (e.g., IOLMaster and Lenstar) to measure AL. Cycloplegic refraction must be performed unless the mean age of the sample was 20 years or older. Spherical equivalent refraction (SER) between −0.50 and +1.25 D was adopted *a priori* as the working definition of emmetropia. The target population was individuals with generally good ocular health (e.g., no history of ocular trauma/surgery, congenital ocular pathology) and between 6–30 years of age.

**FIGURE 1:**
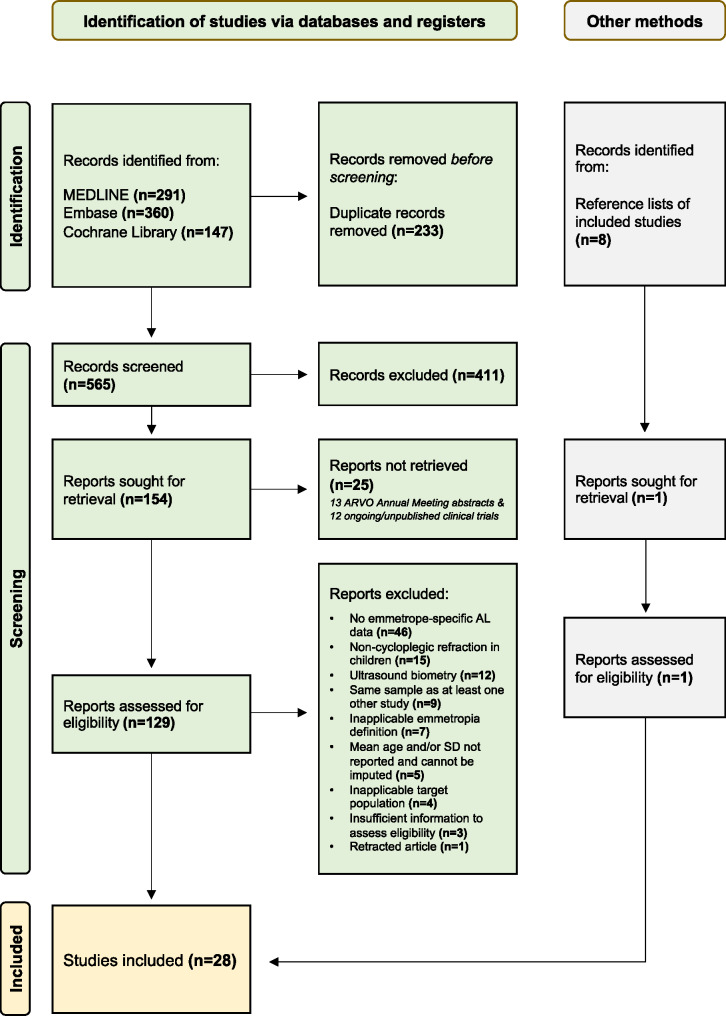
PRISMA flow diagram. From the 129 studies that entered full-text screening, 46 were removed due to the absence of emmetrope-specific axial length (AL) data, 15 were removed due to the use of non-cycloplegic refraction in children aged 20 years or below, 12 were removed due to the use of ultrasonography to measure AL, 9 were removed due to the use of the same sample as at least one other included study, 7 were removed because the definition of emmetropia was outside the range of −0.50 to +1.25 D (e.g., −1.25 to +1.25 D), 4 were removed because the characteristics of the study participants did not match the target population (e.g., younger than 6 years of age) and 3 were removed because there was insufficient information to assess their eligibility (e.g., whether cycloplegic refraction was performed). One retracted article was further removed.

Three electronic databases (MEDLINE, Embase and Cochrane Library) were searched, while restricting the publication language to English, and using search terms that included “emmetropia”, “child“, “young”, “teen”, “adolescent”, “ocular”, “eye”, “axial” and “length”. Twenty eight studies (one identified through searching the reference lists of eligible studies) with subjects having a mean age between 6.5 and 23.1 years were included. More details are provided in the PRISMA flow diagram (Figure 1). Most studies (*n* = 20; 71%) defined emmetropia as ±0.50 D SER, while the remainder defined emmetropia as ±0.50 D spherical power (*n* = 1), −0.25 to +0.50 D SER (*n* = 2), −0.25 to +0.75 D SER (*n* = 1), −0.25 to +1.00 D SER (*n* = 1), −0.50 to +0.75 D SER (*n* = 1), −0.50 to +1.00 D SER (*n* = 1) and −0.50 to +1.25 D SER (*n* = 1). There were 12 non-EA studies (1071 eyes) from Turkey (*n* = 3), UK (*n* = 2), Spain (*n* = 2), Australia (*n* = 2), Norway (*n* = 1), Sweden (*n* = 1) and Denmark (*n* = 1), whereas 16 studies (3331 eyes) were conducted in East Asia, including 12 from Mainland China, 2 from Hong Kong, 1 from South Korea and 1 from Singapore. Full search strategies and characteristics of included studies (along with the complete reference list) can be found in Appendix S1–S3.

Where necessary, the mean ± standard deviation (SD) of a continuous variable of interest was imputed from its median, sample size, interquartile range and/or range.^8^ Where emmetrope-specific mean ± SD age was not reported, the data for the whole sample were used on the condition that the age range was not larger than 3 years. The AL, however, must be specific to emmetropes and not be imputed from the whole sample. Using a weighted nonlinear mixed-effects model, the AL growth curve (mean AL vs. mean age) was first fitted to the full dataset (details of model selection can be found in Appendix S4). The model has a general asymptotic expression as follows:

1 $$ \mathrm{AL}=a+\frac{b}{e^{c\times \mathrm{age}.}} $$

where parameter *a* represents the final AL (horizontal asymptote of the growth curve), parameter *b* represents the amount that the final AL needs to be offset by to obtain AL at age 0 (*y*-intercept), parameter *c* relates to the nonlinear growth rate (curve steepness) and *e* denotes Euler's number (exponential constant equal to 2.718). Study weight (*W*) was computed to give greater importance to studies with lower standard errors (SE) of AL and age, effectively giving studies with higher precision (large sample size and small age range) more influence on parameter estimates:

2 $$ W=\frac{1}{\operatorname{norm}\left(\mathrm{SE}\ \mathrm{of}\ \mathrm{AL}\right)+\operatorname{norm}\left(\mathrm{SE}\ \mathrm{of}\ \mathrm{age}\right)+0.2} $$

where ‘norm’ refers to min–max normalisation; that is, rescaling both SE of AL and SE of age to 0–1 to ensure they have similar influence on *W*. In addition, 0.2 was added to the denominator for numerical stability (prevent division by zero).

The model was then refitted with ethnicity as a two-level grouping variable (EA vs. non-EA), treating non-EA as the reference level:

3 $$ \mathrm{AL}={a}_{\mathrm{nonEA}}+a+\frac{b_{\mathrm{nonEA}}+b}{e^{c_{\mathrm{nonEA}}+c\times \mathrm{age}}} $$

where parameter estimates in non-EA emmetropes are represented by the subscript 'nonEA', while the corresponding original form of the parameter (i.e., without the subscript) denotes the difference between ethnic groups. To illustrate, ${c}_{\mathrm{nonEA}}$ represents the AL growth rate in non-EA emmetropes, while $c$ represents the difference in growth rate between EA and non-EA emmetropes. The AL growth rate in EA emmetropes, by extension, is given by the sum of ${c}_{\mathrm{nonEA}}$ and $c$. Parameter differences between ethnic groups (e.g., $c$) were tested with the Wald test.

## RESULTS AND DISCUSSION

**FIGURE 2:**
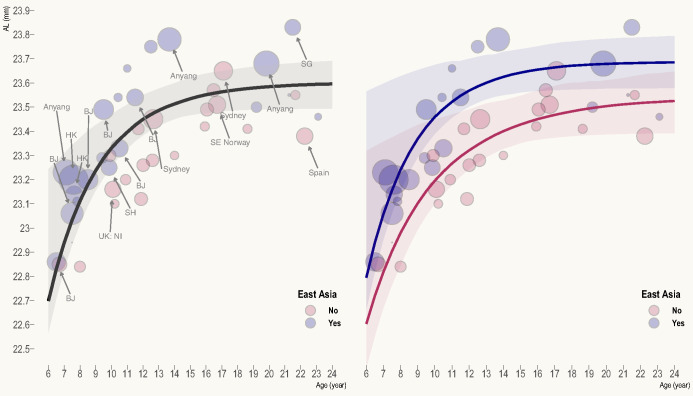
Combined (left) and ethnic-specific (right) axial length (AL) growth curves. Bubble size corresponds to study weight. Studies with large weight (>1.5) are annotated, where SH refers to Shanghai, HK refers to Hong Kong, BJ refers to Beijing, NI refers to Northern Ireland, SE Norway refers to South-eastern Norway, SG refers to Singapore and Anyang refers to Anyang City in Mainland China.

Figure 2 displays the combined and ethnic-specific AL growth curves. They are parametrised as follows:

4 $$ \mathrm{Combined}:\mathrm{AL}=23.60-\frac{5.60}{e^{0.30\times \mathrm{age}}} $$

5 $$ \mathrm{EA}:\mathrm{AL}=23.69-\frac{6.91}{e^{0.33\times \mathrm{age}}} $$

6 $$ \mathrm{Non}-\mathrm{EA}:\mathrm{AL}=23.54-\frac{4.15}{e^{0.24\times \mathrm{age}}} $$

Table 1 summarises the age-specific AL and annual AL growth rate. Across ethnic groups, the annual growth rate decreases from 0.24 mm/year at age 6 to around 0.05 mm/year at age 11, after which it dips below the repeatability of optical biometry (around ±0.04 mm)^6^ and is of little practical importance. Rozema^5^ recently pooled myriad ocular biometric data from 294 studies, including eyes from the foetal period (preterm birth used as surrogate) to 20 years after birth, but without stratification by ethnicity. Primary studies that used participants above 7 years of age were carefully screened in that work to minimise the influence of myopia. The author found that AL growth followed a bi-exponential pattern (two phases), with the first and second phases concluding around 4.5 months and 16 years after birth, respectively. Of note, the final AL (23.60 mm) estimated by the growth curve in the present work, beginning to be reached after age 15, is in close agreement with that derived from Rozema's model (23.61 mm).

**TABLE 1:** Age-specific axial length (AL) and annual growth rate derived from the ethnic-specific and combined growth curves.

Age (years)	Combined		EA		Non-EA	
	AL [95% CI] (mm)	Growth rate (mm/year)	AL [95% CI] (mm)	Growth rate (mm/year)	AL [95% CI] (mm)	Growth rate (mm/year)
6	22.70 [22.57, 23.24]	0.24	22.80 [22.66, 23.56]	0.26	22.60 [22.42, 23.32]	0.22
7	22.94 [22.83, 23.31]	0.17	23.06 [22.95, 23.59]	0.18	22.82 [22.67, 23.36]	0.16
8	23.11 [23.02, 23.37]	0.13	23.24 [23.15, 23.62]	0.13	22.98 [22.85, 23.39]	0.12
9	23.24 [23.16, 23.43]	0.09	23.37 [23.29, 23.65]	0.09	23.10 [23.99, 23.43]	0.10
10	23.33 [23.25, 23.47]	0.07	23.46 [23.39, 23.68]	0.06	23.20 [23.10, 23.45]	0.07
11	23.40 [23.33, 23.51]	0.05	23.52 [23.46, 23.69]	0.05	23.27 [23.18, 23.47]	0.05
12	23.45 [23.38, 23.55]	0.04	23.57 [23.50, 23.70]	0.03	23.32 [23.24, 23.49]	0.04
13	23.49 [23.42, 23.58]	0.03	23.60 [23.53, 23.72]	0.03	23.37 [23.29, 23.52]	0.04
14	23.51 [23.45, 23.60]	0.02	23.63 [23.55, 23.73]	0.02	23.40 [23.33, 23.53]	0.03
15	23.54 [23.47, 23.62]	0.02	23.64 [23.56, 23.74]	0.01	23.43 [23.35, 23.55]	0.02
16	23.55 [23.48, 23.64]	0.01	23.66 [23.57, 23.75]	0.01	23.45 [23.37, 23.57]	0.02
17	23.56 [23.48, 23.65]	0.01	23.66 [23.57, 23.76]	0.01	23.47 [23.37, 23.59]	0.01
18	23.57 [23.49, 23.66]	0.01	23.67 [23.57, 23.77]	0.01	23.48 [23.38, 23.60]	0.01
19	23.58 [23.49, 23.67]	0.01	23.68 [23.58, 23.78]	0.00	23.49 [23.38, 23.61]	0.01
20	23.58 [23.49, 23.67]	0.00	23.68 [23.58, 23.78]	0.00	23.50 [23.39, 23.61]	0.01
21	23.59 [23.49, 23.68]	0.00	23.68 [23.58, 23.79]	0.00	23.51 [23.39, 23.62]	0.01
22	23.59 [23.49, 23.68]	0.00	23.68 [23.58, 23.79]	0.00	23.52 [23.39, 23.63]	0.00
23	23.59 [23.49, 23.69]	0.00	23.68 [23.58, 23.79]	0.00	23.52 [23.39, 23.64]	0.00
24	23.60 [23.49, 23.69]	0.00	23.69 [23.58, 23.79]	0.00	23.53 [23.39, 23.65]	0.00

Abbreviations: CI, confidence interval; EA, East Asians.

### Comparison between ethnic groups

There is no evidence that the AL growth rate (as indicated by curve steepness) differs between EA and non–EA emmetropes (difference in parameter *c*: 0.09, 95% CI: −0.13 to 0.31, *p* = 0.43). It is generally accepted that disparities in environmental exposures (e.g., higher engagement in education among EA children), rather than genetic factors per se, give rise to differences in myopia prevalence across ethnicities.^9^ Given the lack of ethnic differences in the underlying genetic predisposition to myopia, and that emmetropes from different ethnic groups can be assumed to have comparably low levels of environmental exposures (as otherwise they would not be emmetropic in the first place), it is perhaps not surprising that an ethnic difference in AL growth rate was absent.

In keeping with Chen et al.,^4^ who reported no significant difference in AL between EA and non-EA children aged 7 years and under (after adjustment for measurement method, i.e., ultrasonography vs. non-contact approach), the present work also found insufficient statistical evidence of a difference in the final AL (difference: 0.15 mm, 95% CI: −0.04 to 0.35 mm, *p* = 0.15) and parameter *b* (difference: −2.77 mm, 95% CI: −10.97 to 5.44, *p* = 0.51) between ethnic groups, although from Figure 2 and Table 1, AL appears to increase from a higher initial value at age 6 (22.80 mm vs. 22.60 mm) to a higher final value at age 24 (23.69 mm vs. 23.53 mm) in EA emmetropes. This apparent systematic offset, however, is attributable to a slight ethnic difference in the underlying SER distribution, given that the proportion of EA studies that defined emmetropia as ±0.50 D SER (81%), which had a lower hyperopic upper limit, was somewhat higher than that of non-EA studies (58%). Indeed, after controlling for refraction by refitting the model to studies that applied a similar definition of emmetropia (±0.50 D SER), the difference in final AL between ethnic groups reduced from 0.15 mm (95% CI: −0.04 to 0.35 mm) to −0.02 mm (95% CI: −0.33 to 0.30 mm). Importantly, there was still no evidence of a significant difference (all *p* > 0.05) in any of the growth curve parameters (including AL growth rate) between EA and non-EA emmetropes.

Compared to males, females are known to have a shorter AL on account of their generally shorter stature (readers may refer to the discussion in Rozema^5^ for a concise overview). Moreover, a general difference in AL growth rate may also exist between female and male emmetropes during childhood/puberty, given that changes in height and AL have been shown to be correlated in young emmetropes.^10^ That said, it is unlikely that sex could have confounded the (null) findings of this work, as the overall sex distribution across studies here did not differ significantly between ethnic groups (53% and 48% of individuals were males across EA and non-EA studies that provided sex breakdown for emmetropes).

### Comparison with persistent emmetropes

**FIGURE 3:**
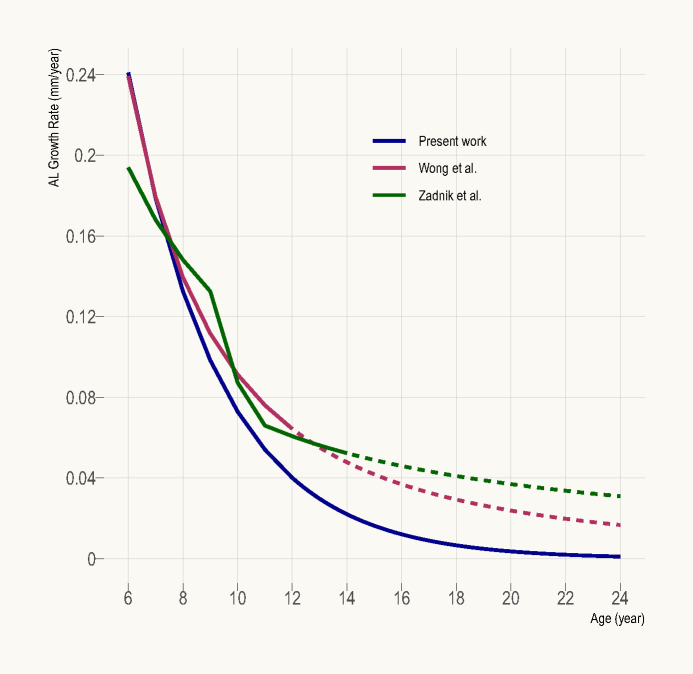
Annual axial length (AL) growth rate as a function of age derived from the present work (blue), Wong et al.^2^ (maroon; rate of change in vitreous chamber depth used as surrogate for AL growth rate because the presented AL growth model unrealistically predicts shrinking AL when extrapolated beyond 12 years old) and Zadnik et al.^3^ (green). Extrapolated data are represented by dotted lines.

One potential limitation of the present work is its limited applicability to persistent emmetropes on account of its use of predominantly cross–sectional data (where status of emmetropia was only confirmed at a single time point). Figure 3 compares the age–specific AL growth rate determined in this work to that derived from two earlier longitudinal studies; Wong et al.^2^ (Asian participants) and Zadnik et al.^3^ (87.5% Caucasian participants), who examined persistent emmetropes aged up to 12 and 14 years, respectively. The former study defined persistent emmetropia as SER between −0.50 and +1.00 D over at least three annual visits, while the latter defined it as spherical power between −0.25 and +1.00 D in both meridians over at least two annual visits. If one supposes that most young emmetropes included in the present work were indeed future myopes, a group known to undergo faster axial growth than persistent emmetropes,^11^ then the growth rate determined herein should be overestimated (higher) relative to Wong et al.^2^ and Zadnik et al.^3^ Contrary to this hypothetical expectation, the present work consistently (to all intents and purposes) predicts a similar or lower growth rate than both studies (Figure 3), suggesting that the present growth curve may in fact be applicable to persistent emmetropes.

## CONCLUSIONS

Normal emmetropes undergo decelerated axial growth from 6 to 11 years of age, after which the growth rate is of little practical significance. There is insufficient evidence that AL growth curve parameters differ between EA and non-EA emmetropes. This suggests that emmetropic AL growth charts, which can be used to predict myopia development by facilitating comparison between a child's AL trajectory and the age-normative data, may be shared or pooled across ethnic groups, although this prospect remains to be validated by future clinical research.

## Supplementary Information

Supplementary file (DOCX 155 KB)

## Data Availability

Extracted data (cleaned) from the primary studies and R code are freely available at https://github.com/fyii200/AL-Meta-Regression.
