# Supplementary material for: Emmetropic eye growth in East Asians and non-East Asians
Source: Ophthalmic Physiol Opt. 2023 Jun 27;43(6):1412–8. doi: 10.1111/opo.13195 (PMC10947352; doi:10.1111/opo.13195)
Supplement: Supplementary file 1 — Supplementary file (DOCX 155 KB) [file 44402_2023_4306012_MOESM1_ESM.docx]

**Supplementary Material**

**S1:** Full search strategies

*MEDLINE (via Ovid interface), Embase (via Ovid interface) and Cochrane Library search strategies are presented below. All searches took place on 21 November 2022. Searches were restricted to 1 January 2000 and thereafter, as the first optical biometer only became commercially available in 1999 and received FDA approval in 2000.^1^*

**MEDLINE(R) and In-Process, In-Data-Review & Other Non-Indexed Citations from 1946 to November 18, 2022:**

1. Emmetropia/ (426)
2. emmetropi*.mp. (3190)
3. 1 or 2 (3190)
4. (child* or young* or teen* or adolescen*).mp. (4679796)
5. 3 and 4 (1374)
6. ((ocular or eye or axial) and length).mp. (22259)
7. 5 and 6 (379)
8. exp animals/ not humans.sh. (5061820)
9. 7 not 8 (357)
10. limit 9 to english language (327)
11. limit 10 to yr="2000 -Current" (291)

**Embase from 1974 to 2022 Week 46:**

1. exp emmetropia/ (1719)
2. emmetropi*.mp. (4423)
3. 1 or 2 (4423)
4. (child* or young* or teen* or adolescen*).mp. (4768316)
5. 3 and 4 (1646)
6. ((ocular or eye or axial) and length).mp. (31688)
7. 5 and 6 (473)
8. (exp animal/ or nonhuman/) not exp human/ (6937843)
9. 7 not 8 (433)
10. limit 9 to english language (388)
11. limit 10 to yr="2000 -Current" (360)

**Cochrane Library: 147 clinical trials from 2000 to 2020**

1. emmetropia
2. emmetrop*
3. #1 OR #2
4. child* OR young* OR teen* OR adolescen*
5. #3 AND #4
6. (ocular OR eye OR axial) AND length
7. #5 AND #6
8. myopia AND (onset or prevent* or develop*)
9. #4 AND #6 AND #8
10. #7 OR #9

**S2:** Characteristics of 28 included studies

| Study | Country | Data source | Cycloplegia protocol | Emm | Ref | OB |
| --- | --- | --- | --- | --- | --- | --- |
|  | | | | | | |
| Little et al.^2^ | UK (Northern Ireland) | School-based population study (Northern Ireland Childhood Errors of Refraction study) | 1 drop of Cyclopentolate 1%; 1 extra drop if cycloplegia not evident after 15 min; refraction after 30 min | ±0.50 | Auto | IOL |
| Barrio-Barrio et al.^3^ | Spain (Madrid, Pamplona, Bilbao & Zaragoza) | Healthy sample from ophthalmology clinic | 3 drops of Cyclopentolate 1% 5 min apart; refraction after 30 min | -0.50 to +1.25 | Auto | IOL |
| Xu et al.^4^ | China (Wenzhou) | Healthy sample (setting unclear) | 3 drops of Cyclopentolate 1% 5 min apart; refraction after 30 min | Sphere ±0.50 | Auto | IOL |
| Martinez et al.^5^ | Australia (Sydney) | School-based population study (Sydney Myopia Study) | 2 cycles of Cyclopentolate 1% (1 drop) & Tropicamide 1% (1 drop) 5 minutes apart; up to 2 drops of Phenylephrine 2.5% as needed; refraction after >30 min | ±0.50 | Auto | IOL |
| Hagen et al.^6^ | Norway (Southeast) | School-based population study | 1 drop of Cyclopentolate 1% (light irides) or 2 drops (dark irides); refraction after 15-20 min | ±0.50 | Auto | IOL |
| Shao et al.^7^ | China (Shenzhen) | Healthy sample from optometry clinic | Limited information: Atropine 1% ointment for 3 days if <12 years old; otherwise, “compound Tropicamide” | -0.25 to +0.50 | Subj? | IOL |
| Bueno-Gimeno et al.^8^ | Spain (Valencia) | Healthy sample from ophthalmology clinic | 3 drops of Cyclopentolate 1% 5 min apart; cycloplegia confirmed if pupil diameter ≥6mm; refraction after 30 min | -0.25 to +0.75 | Auto | IOL |
| Sun et al.^9^ | China (Anyang) | University-based population study (Anyang University Students Eye study) | 1 drop of Cyclopentolate 1% & 1 drop of Tropicamide 0.5% + Phenylephrine 0.5% compound (5 min apart); 1 extra drop of cyclopentolate 1% if pupillary light reflex was present/ pupil size<6mm; refraction after 30 min | ±0.50 | Auto | Len |
| Li et al.^10^ | China (Beijing) | Healthy sample from ophthalmology clinic | Limited information other than saying “under cycloplegia” | ±0.50 | Auto | IOL |
| Ehsaei et al.^11^ | UK (Bradford) | Healthy university student population | Cycloplegics not used | ±0.50 | Subj | IOL |
| Aykut et al.^12^ | Turkey (?) | Healthy sample (setting unclear) | 3 drops of Cyclopentolate 1% 5 min apart; refraction after 30 min | -0.25 to +0.50 | Auto | IOL |
| Fledelius et al.^13^ | Denmark (Copenhagen?) | Healthy (included eye) sample from ophthalmology clinic | Limited information other than saying “refractive state was determined subjectively, usually after dilation with Mydriacyl (i.e. Tropicamide) 0.5% eye drops” | -0.25 to +1.00 | Subj | IOL |
| Philip et al.^14^ | Australia (Sydney) | Healthy sample from a school-based population study (Sydney Myopia Study from 2004-05, and then Sydney Adolescent Vascular and Eye Study from 2009-10) | 2 cycles of Cyclopentolate 1% (1 drop) & Tropicamide 1% (1 drop) 5 minutes apart; up to 2 drops of Phenylephrine 2.5% as needed; refraction after >30 min | ±0.50 | Auto | IOL |
| Li et al.^15^ | China (Anyang) | School-based population study (Anyang Childhood Eye Study); data from 14-year-olds used in meta-regression | 2 drops of Cyclopentolate 1% followed by 1 drop of Tropicamide 0.5%; refraction after 30 min | ±0.50 | Auto | Len |
| Li et al.^16^ | China (Anyang) | School-based population study (Anyang Childhood Eye Study); data from 7-year-olds used in meta-regression | 2 drops of Cyclopentolate 1% followed by 1 drop of Tropicamide 0.5%; refraction after 30 min | ±0.50 | Auto | Len |
| Bulut et al.^17^ | Turkey (Rize?) | Healthy sample from ophthalmology clinic | 3 drops of Cyclopentolate 1% 10 min apart | ±0.50 | ? | Len |
| Jin et al.^18^ | China (Shanghai) | Healthy sample from a school-based population study | 2 drops of Cyclopentolate 1% 5 min apart; 1 extra drop if pupillary light reflex was still present and pupil diameter<6mm after 30 min | ±0.50 | Auto | IOL |
| Li et al.^19^ | China (Hong Kong) | Healthy sample (from optometry clinic?) | 2 drops of Tropicamide 0.5% 5 min apart; refraction after 30 min | ±0.50 | Subj | IOL |
| Sung et al.^20^ | South Korea (Gwangju) | Healthy sample (from ophthalmology clinic ?) | Cycloplegics not used | ±0.50 | Subj | Len |
| Chen et al.^21^ | Singapore | Healthy sample from a compulsory pre-employment eye screening programme | 3 drops of Cyclopentolate 1% 5 min apart; refraction after 30 min | -0.50 to +1.00 | Subj | IOL |
| Dogan et al.^22^ | Turkey (Ankara) | Healthy sample (routine eye exams) | 3 drops of Cyclopentolate 1% 5 min apart; refraction after 30 min | ±0.50 | Auto | Len |
| Duan et al.^23^ | China (Guangzhou) | Healthy medical students | 3 drops of Tropicamide 1% | ±0.50 | Auto | Len |
| Li et al.^24^ | China (Hong Kong) | School-based population study (Hong Kong Children Eye Study) | 2 drops of Cyclopentolate 1% 5 min apart (repeated after 10 min); further cycles repeated if pupillary light reflex was present/ if pupil diameter<6mm; refraction after 30 min | ±0.50 | Auto | IOL |
| Demir et al.^25^ | Sweden (Kalmar) | Healthy sample from a school-based population study | 2 drops of Cyclopentolate 1% 10 min apart; refraction after 30 min | -0.50 to +0.75 | Auto | IOL |
| Jin et al.^26^ | China (Tianjin) | Healthy sample from optometry clinic | 3 drops of Cyclopentolate 1% 5 min apart; refraction after 45 min | ±0.50 | Auto | Len |
| Zheng et al.^27^ | China (Guangzhou) | Sample from ophthalmology clinic | 2 drops of Tropicamide + Phenylephrine (ambiguous dose) compound; refraction after 30 min | ±0.50 | Auto | Len |
| Lv et al.^28^ | China (Wuhan) | Healthy sample from optometry clinic | Cyclopentolate 1% (number of drops unclear); refraction after 25-30 min | ±0.50 | Auto | IOL |
| Li et al.^29^ | China (Beijing) | School-based population study (Beijing Pinggu Childhood Eye Study) | 2 drops of Cyclopentolate 1% 5 min apart, with a third drop instilled after 20 min; refraction after 15 min if no pupillary light reflex and pupil diameter ≥ 6mm | ±0.50 | Auto | IOL |
|  | | | | | | |
| Emm=definition of emmetropia (SER range unless otherwise stated); Ref=refraction method; OB=optical biometer; Auto=autorefraction; Subj=subjective refraction; IOL=IOLMaster; Len=Lenstar | | | | | | |

**S3:** Pertinent data extracted from each study

| Study | N | Male % | Age range | Mean age | SD of age | Mean AL | SD of AL | Mean SER (SD) | W |
| --- | --- | --- | --- | --- | --- | --- | --- | --- | --- |
|  |  | | | | | | | | |
| *East Asia* |  | | | | | | | | |
| Li et al.^29^ | 59 | ? | 6 | 6.5 | 0.15 | 22.86 | 0.55 | ? | 2.0 |
| Li et al.^16^ | 318 | 58 | 7 | 7.1 | 0.4 | 23.23 | 0.62 | +0.11 (0.24) | 3.6 |
| Li et al.^29^ | 146 | ? | 7 | 7.5 | 0.15 | 23.06 | 0.60 | ? | 2.8 |
| Shao et al.^7^ | 6 | 50 | 5-10 | 7.5 | 2.3 | 22.94 | 0.48 | +0.06 (0.21) | 0.5 |
| Li et al.^24^ | 866 | 47 | 5-10 | 7.6 | 0.9 | 23.20 | 0.66 | +0.09 (0.26) | 4.6 |
| Li et al.^19^ | 56 | 48 | 6-9 | 7.6 | 0.46 | 23.14 | 0.60 | +0.19 (0.33) | 1.7 |
| Xu et al.^4^ | 22 | 59 | 5-12 | 7.8 | 1.1 | 23.11 | 0.81 | -0.02 (0.28) | 0.8 |
| Li et al.^29^ | 112 | ? | 8 | 8.5 | 0.15 | 23.20 | 0.63 | ? | 2.4 |
| Jin et al.^26^ | 38 | 66 | 4-15 | 9.4 | 2.5 | 23.29 | 0.62 | -0.08 (0.26) | 1.0 |
| Li et al.^29^ | 112 | ? | 9 | 9.5 | 0.15 | 23.49 | 0.65 | ? | 2.4 |
| Jin et al.^18^ | 91 | 65 | 7-13 | 9.9 | 1.2 | 23.25 | 0.72 | +0.18 (0.26) | 1.6 |
| Li et al.^10^ | 22 | 46 | 8-16 | 10.4 | 1.7 | 23.54 | 0.74 | -0.05 (0.37) | 0.8 |
| Li et al.^29^ | 89 | ? | 10 | 10.5 | 0.15 | 23.33 | 0.75 | ? | 1.9 |
| Lv et al.^28^ | 22 | 45 | ? | 11.0 | 2.2 | 23.66 | 0.74 | +0.03 (0.13) | 0.7 |
| Li et al.^29^ | 49 | ? | 11 | 11.5 | 0.15 | 23.54 | 0.59 | ? | 1.8 |
| Li et al.^29^ | 50 | ? | 12 | 12.5 | 0.15 | 23.75 | 0.93 | ? | 1.2 |
| Li et al.^15^ | 327 | 54 | 14 | 13.7 | 0.5 | 23.78 | 0.75 | +0.01 (0.33) | 3.1 |
| Duan et al.^23^ | 22 | 59 | 17-23 | 19.2 | 0.6 | 23.50 | 0.70 | +0.3 (0.6) | 1.0 |
| Sun et al.^9^ | 721 | 44 | 17-24 | 19.8 | 1.5 | 23.68 | 0.79 | -0.01 (0.26) | 3.5 |
| Zheng et al.^27^ | 21 | 48 | 18-28 | 21.3 | 4.8 | 23.55 | 0.74 | -0.28 (0.21) | 0.6 |
| Chen et al.^21^ | 156 | 100 | 19-25 | 21.5 | 1.1 | 23.83 | 1.01 | +0.40 (0.39) | 1.6 |
| Sung et al.^20^ | 26 | 50 | 20-30 | 23.1 | 4.3 | 23.46 | 0.55 | -0.08 (0.38) | 0.7 |
|  |  | | | | | | | | |
|  |  | | | | | | | | |
| *Outside East Asia* |  | | | | | | | | |
| Martinez et al.^5^ | 51 | ? | 6 | 6.7 | 0.4 | 22.85 | 0.71 | +0.20 (0.26) | 1.5 |
| Fledelius et al.^13^ | 21 | 57 | 7-9 | 8.0 | 0.5 | 22.84 | 0.65 | +0.54 (0.45) | 1.0 |
| Fledelius et al.^13^ | 29 | 45 | 9-11 | 9.9 | 0.6 | 23.30 | 0.67 | +0.28 (0.49) | 1.1 |
| Little et al.^2^ | 46 | ? | 9-10 | 10.1 | 0.4 | 23.16 | 0.56 | +0.06 (0.29) | 1.7 |
| Aykut et al.^12^ | 40 | 55 | 6-14 | 10.2 | 1.8 | 23.10 | 1.00 | +0.24 (0.0) | 0.8 |
| Bulut et al.^17^ | 64 | 44 | 5-17 | 10.9 | 3.4 | 23.20 | 0.80 | +0.0 (0.3) | 1.0 |
| Dogan et al.^22^ | 50 | 56 | 6-16 | 11.7 | 2.6 | 23.41 | 0.61 | -0.08 (0.49) | 1.1 |
| Bueno-Gimeno et al.^8^ | 99 | ? | 6-17 | 11.9 | 3.0 | 23.12 | 0.79 | +0.34 (0.41) | 1.2 |
| Fledelius et al.^13^ | 33 | 70 | 11-13 | 12.0 | 0.6 | 23.26 | 0.68 | +0.28 (0.22) | 1.2 |
| Demir et al.^25^ | 54 | ? | 8-16 | 12.6 | 2.0 | 23.28 | 0.67 | ? | 1.2 |
| Philip et al.^14^ | 125 | ? | 12 | 12.7 | 0.5 | 23.45 | 0.68 | +0.20 (0.23) | 2.2 |
| Fledelius et al.^13^ | 23 | 55 | 13-15 | 14.0 | 0.6 | 23.30 | 0.93 | +0.16 (0.13) | 0.8 |
| Fledelius et al.^13^ | 14 | 71 | 13-15 | 15.9 | 0.5 | 23.42 | 0.65 | +0.11 (0.31) | 0.8 |
| Little et al.^2^ | 39 | ? | 15-16 | 16.1 | 0.3 | 23.49 | 0.75 | +0.13 (0.39) | 1.3 |
| Barrio-Barrio et al.^3^ | 52 | 52 | 13-17 | 16.5 | 1.2 | 23.57 | 0.73 | +0.07 (0.46) | 1.2 |
| Hagen et al.^6^ | 120 | ? | 16-19 | 16.7 | 0.9 | 23.51 | 0.75 | +0.18 (0.23) | 1.9 |
| Philip et al.^14^ | 125 | ? | 17 | 17.1 | 0.7 | 23.65 | 0.71 | +0.15 (0.33) | 2.1 |
| Fledelius et al.^13^ | 21 | 43 | 17-20 | 18.6 | 1.0 | 23.41 | 0.74 | +0.19 (0.26) | 0.8 |
| Ehsaei et al.^11^ | 27 | ? | ? | 21.7 | 3.2 | 23.55 | 0.48 | +0.10 (0.31) | 0.8 |
| Barrio-Barrio et al.^3^ | 163 | 37 | 18-28 | 22.3 | 2.6 | 23.38 | 0.72 | +0.05 (0.45) | 1.7 |
|  |  | | | | | | | | |
| N=total number of emmetropic eyes; Male %=percentage of male emmetropic eyes; W=study weight  Li et al.^29^ Age-specific (i.e. rounded age corresponding to 6, 7, 8….12 years old) data provided in the paper. Mean±SD age for each age group is imputed by assuming normal distributions centred at 6.5, 7.5, 8.5….12.5, with the SD being 0.15 for each distribution, such that for any given age interval (6-7y/o for example) >99% of data will lie between the age interval (e.g. 6.05 and 6.95 y/o with a mean±SD of 6.5±0.15).  Shao et al.^7^ Emmetrope-specific AL data are computed from the individual-level raw data (provided as Supporting Information).  Li et al.,^24^ Jin et al.,^26^ Chen et al.,^21^ Duan et al.,^23^ Li et al.,^10^ Jin et al.^18^ & Zheng et al.^27^ age range is imputed from the whole sample which includes some non-emmetropes.  Li et al.^19^ SD of age is imputed from age range, i.e. with a SD of 0.46, the lower and upper limits (3 standard deviations; correspond to 6.2 to 9.0) of the age distribution (assumed normality) will be consistent with the observed range of 6-9.  Sun et al.^9^ emmetrope-specific mean±SD age is reported in another study* that uses the same dataset.  Fledelius et al.^13^ mean±SD age computed based on the extracted age values from Figure 1.  Hagen et al.^6^ mean±SD age is imputed from the whole sample which includes non-emmetropes.  Little et al.^2^ mean±SD AL and mean±SD SER are imputed from their respective median and interquartile range provided in Table 1. In addition, mean±SD age is imputed from the whole sample which includes non-emmetropes.  **Wei S, Sun Y, Li S, Hu J, Yang X, Lin C et al. Refractive Errors in University Students in Central China: The Anyang University Students Eye Study. Invest Ophthalmol Vis Sci 2018; 59(11): 4691-4700.* | | | | | | | | | |

**S4:** Details of model selection

Four candidate weighted nonlinear mixed-effects models were fitted to the full dataset using maximum likelihood to describe mean AL as a function of mean age. These models can be expressed as follows:

*AL =* $a+ \frac{b}{e^{c \times age}}$ **(1)**

*AL =* $a+ \frac{1}{e^{c \times age}}$ **(2)**

*AL =* $a+ \frac{b}{e^{age}}$ **(3)**

*AL =*$a+ \frac{b}{age}$ **(4)**

Likelihood-ratio test was performed to compare the fits of the four nonlinear models. The model with the highest log-likelihood (beyond what one would expect by chance alone) was chosen as the final model. Akaike Information Criterion (AIC) and Bayesian Information Criterion (BIC) were also used to guide model selection, i.e. lower score (parsimonious model: simple model with great predictive power) was preferred.

Model (1) had significantly higher log-likelihood than other nonlinear models (Table 1). A visual comparison of the 4 fitted models is presented in Figure 1, which also shows that model (1) has the best fit.

Table 1 Likelihood ratio test

| Comparisons | Parameters [95% CI] | No. of parameters | AIC | BIC | Log likelihood | Df | Chi^2^ | P |
| --- | --- | --- | --- | --- | --- | --- | --- | --- |
| *Model (2)* | **a**= 21.99, **c**= −0.03 | 4 | −9.83 | −2.88 | 8.92 | 1 | 27.50 | <0.001 |
| *Model (1)* | **a**= 23.60, **b**= −5.60, **c**= 0.30 | 5 | −35.33 | −26.64 | 22.67 |  |  |  |
| *Model (3)* | **a**= 23.45, **b**= −515.51 | 4 | −14.32 | −7.37 | 11.16 | 1 | 23.01 | <0.001 |
| *Model (1)* | **a**= 23.60, **b**= −5.60, **c**= 0.30 | 5 | −35.33 | −26.64 | 22.67 |  |  |  |
| *Model (4)* | **a**= 24.03, **b**= −7.55 | 4 | −31.42 | −24.47 | 19.71 | 1 | 5.91 | 0.015 |
| *Model (1)* | **a**= 23.60, **b**= −5.60, **c**= 0.30 | 5 | −35.33 | −26.64 | 22.67 |  |  |  |

Figure 1 Four candidate nonlinear models fitted to the full dataset. The best-fitting model is represented by the black curve (corresponds to model 1), whereas blue, maroon and orange curves correspond to Model 2, Model 3 and Model 4, respectively.

**References**

1. Vogel A, Dick HB, Krummenauer F. Reproducibility of optical biometry using partial coherence interferometry : intraobserver and interobserver reliability. J Cataract Refract Surg. 2001;27(12):1961-8.

2. Little JA, McCullough SJ, Breslin KM, Saunders KJ. Higher order ocular aberrations and their relation to refractive error and ocular biometry in children. Invest Ophthalmol Vis Sci. 2014;55(8):4791-800. Epub 20140715.

3. Barrio-Barrio J, Bonet-Farriol E, Galdós M, Noval S, Pueyo V, Breeze CE, et al. HGF-rs12536657 and Ocular Biometric Parameters in Hyperopic Children, Emmetropic Adolescents, and Young Adults: A Multicenter Quantitative Trait Study. J Ophthalmol. 2019;2019:7454250. Epub 20190203.

4. Xu J, Zheng J, Yu S, Sun Z, Zheng W, Qu P, et al. Macular choroidal thickness in unilateral amblyopic children. Invest Ophthalmol Vis Sci. 2014;55(11):7361-8. Epub 20141014.

5. Martinez AA, Sankaridurg PR, Naduvilath TJ, Mitchell P. Monochromatic aberrations in hyperopic and emmetropic children. J Vis. 2009;9(1):23.1-14. Epub 20090120.

6. Hagen LA, Gjelle JVB, Arnegard S, Pedersen HR, Gilson SJ, Baraas RC. Prevalence and Possible Factors of Myopia in Norwegian Adolescents. Sci Rep. 2018;8(1):13479. Epub 20180907.

7. Shao X, Zou C, Qin B. Correlation of choroidal thickness and ametropiain young adolescence. PLoS One. 2017;12(4):e0174385. Epub 20170412.

8. Bueno-Gimeno I, España-Gregori E, Gene-Sampedro A, Ondategui-Parra JC, Zapata-Rodriguez CJ. Variations of OCT measurements corrected for the magnification effect according to axial length and refractive error in children. Journal of Innovative Optical Health Sciences. 2018;11(1):1850001.

9. Sun Y, Wei S, Li S, Cao K, Hu J, Yang X, et al. Distribution of ocular biometry in young Chinese eyes: The Anyang University Students Eye Study. Acta Ophthalmol. 2021;99(6):621-7. Epub 20201216.

10. Li J, Zhu L, Zhu R, Lu Y, Rong X, Zhang Y, et al. Automated Analysis of Choroidal Sublayer Morphologic Features in Myopic Children Using EDI-OCT by Deep Learning. Transl Vis Sci Technol. 2021;10(13):12.

11. Ehsaei A, Chisholm CM, Pacey IE, Mallen EA. Off-axis partial coherence interferometry in myopes and emmetropes. Ophthalmic Physiol Opt. 2013;33(1):26-34. Epub 20121121.

12. Aykut V, Öner V, Taş M, Işcan Y, Ağaçhan A. Influence of axial length on peripapillary retinal nerve fiber layer thickness in children: a study by RTVue spectral-domain optical coherence tomography. Curr Eye Res. 2013;38(12):1241-7. Epub 20130823.

13. Fledelius HC, Christensen AS, Fledelius C. Juvenile eye growth, when completed? An evaluation based on IOL-Master axial length data, cross-sectional and longitudinal. Acta Ophthalmol. 2014;92(3):259-64. Epub 20130410.

14. Philip K, Sankaridurg P, Holden B, Ho A, Mitchell P. Influence of higher order aberrations and retinal image quality in myopisation of emmetropic eyes. Vision Res. 2014;105:233-43. Epub 20141111.

15. Li SM, Iribarren R, Kang MT, Li H, Li SY, Liu LR, et al. Corneal Power, Anterior Segment Length and Lens Power in 14-year-old Chinese Children: the Anyang Childhood Eye Study. Sci Rep. 2016;6:20243. Epub 20160201.

16. Li SM, Wang N, Zhou Y, Li SY, Kang MT, Liu LR, et al. Paraxial Schematic Eye Models for 7- and 14-Year-Old Chinese Children. Invest Ophthalmol Vis Sci. 2015;56(6):3577-83.

17. Bulut A, Öner V, Büyüktarakçı Ş, Kaim M. Associations between choroidal thickness, axial length and spherical equivalent in a paediatric population. Clin Exp Optom. 2016;99(4):356-9. Epub 20160506.

18. Jin P, Zou H, Zhu J, Xu X, Jin J, Chang TC, et al. Choroidal and Retinal Thickness in Children With Different Refractive Status Measured by Swept-Source Optical Coherence Tomography. Am J Ophthalmol. 2016;168:164-76. Epub 20160514.

19. Li SZ, Yu WY, Choi KY, Lam CH, Lakshmanan Y, Wong FS, et al. Subclinical Decrease in Central Inner Retinal Activity Is Associated With Myopia Development in Children. Invest Ophthalmol Vis Sci. 2017;58(10):4399–406.

20. Sung MS, Lee TH, Heo H, Park SW. Association Between Optic Nerve Head Deformation and Retinal Microvasculature in High Myopia. Am J Ophthalmol. 2018;188:81-90. Epub 20180206.

21. Chen DZ, Koh V, Tan M, Tan CS, Nah G, Shen L, et al. Peripheral retinal changes in highly myopic young Asian eyes. Acta Ophthalmol. 2018;96(7):e846-e51. Epub 20180325.

22. Dogan M, Elgin U, Sen E, Tekin K, Yilmazbas P. Comparison of anterior segment parameters and axial lengths of myopic, emmetropic, and hyperopic children. Int Ophthalmol. 2019;39(2):335-40. Epub 20171229.

23. Duan F, Yuan Z, Deng J, Wong YL, Yeo AC, Chen X. Choroidal Thickness and Associated Factors among Adult Myopia: A Baseline Report from a Medical University Student Cohort. Ophthalmic Epidemiol. 2019;26(4):244-50. Epub 20190408.

24. Li FF, Lu SY, Tang SM, Kam KW, Pancy O S T, Yip WWK, et al. Genetic associations of myopia severities and endophenotypes in children. Br J Ophthalmol. 2021;105(8):1178-83. Epub 20200814.

25. Demir P, Baskaran K, Theagarayan B, Gierow P, Sankaridurg P, Macedo AF. Refractive error, axial length, environmental and hereditary factors associated with myopia in Swedish children. Clin Exp Optom. 2021;104(5):595-601. Epub 20210302.

26. Jin CC, Pei RX, Du B, Liu GH, Jin N, Liu L, et al. Lag of accommodation predicts clinically significant change of spherical equivalents after cycloplegia. Int J Ophthalmol. 2021;14(7):1052-8. Epub 20210718.

27. Zheng X, Cheng D, Lu X, Yu X, Huang Y, Xia Y, et al. Relationship Between Peripheral Refraction in Different Retinal Regions and Myopia Development of Young Chinese People. Front Med (Lausanne). 2021;8:802706. Epub 20220118.

28. Lv L, Li M, Chang X, Zhu M, Liu Y, Wang P, et al. Macular Retinal Microvasculature of Hyperopia, Emmetropia, and Myopia in Children. Front Med (Lausanne). 2022;9:900486. Epub 20220520.

29. Li Y, Xing Y, Jia C, Ma J, Li X, Zhou J, et al. Beijing Pinggu Childhood Eye Study: The Baseline Refractive Characteristics in 6- to 12-Year-Old Chinese Primary School Students. Front Public Health. 2022;10:890261. Epub 20220527.
